# Metabolomic analysis of synovial fluid from healthy and pathological equine joints and tendon sheaths using high-resolution ^1^H Nuclear Magnetic Resonance

**DOI:** 10.3389/fvets.2025.1671176

**Published:** 2025-12-16

**Authors:** Marta Guadalupi, Chiara Roberta Girelli, Simone Della Tommasa, Federica Della Corte, Alberto Maria Crovace, Francesco Paolo Fanizzi, Walter Brehm, Luca Lacitignola

**Affiliations:** 1 Dipartimento di Medicina di Precisione e Rigenerativa e Area Jonica, Università degli Studi di Bari Aldo Moro, Bari, Italy; 2 Department of Biological and Environmental Sciences and Technologies, University of Salento, Lecce, Italy; 3 Department for Horse, University of Leipzig, Leipzig, Germany; 4 Dipartimento di Medicina Veterinaria, Università di Sassari, Sassari, Italy

**Keywords:** horse, synovial fluid, joint, tendon sheath, biomarker, ^1^H-Nuclear Magnetic Resonance spectroscopy

## Abstract

**Introduction:**

Joint and tendon sheath diseases are a major cause of lameness and reduced performance in horses. Synovial fluid composition changes in response to pathological processes and metabolomic profiling offers a promising approach to detect these alterations. While equine joint metabolomics has been explored, little is known about the metabolomic profile of tendon sheaths. This study aimed to characterize and compare the synovial fluid metabolomic profiles of healthy and pathological joints and tendon sheaths in horses using high-resolution ^1^H Nuclear Magnetic Resonance spectroscopy, and to identify potential biomarkers associated with musculoskeletal pathology.

**Methods:**

Synovial fluid samples were collected from healthy joints and tendon sheaths of routinely slaughtered animals, and from pathological joints and tendon sheaths from owned athletic horses affected by inflammatory or degenerative conditions. The samples were analyzed using ^1^H Nuclear Magnetic Resonance spectroscopy. Synovial fluid samples were collected from healthy joints and tendon sheaths of routinely slaughtered animals, and from pathological joints and tendon sheaths from owned athletic horses affected by inflammatory or degenerative conditions. The samples were analyzed using ^1^H Nuclear Magnetic Resonance spectroscopy.

**Results:**

The metabolomic analysis of equine synovial fluid identified amino acids, organic acids, glucose isomers, and other metabolites. No significant differences were observed in the metabolic profiles of synovial fluid from healthy joints and tendon sheaths (PCA: R^2^X = 0.761, Q2 = 0.372; OPLS-DA: R^2^X = 0.48; R^2^Y = 0.292; Q2 = −0.143). In contrast, a clear separation with distinct clustering was observed between healthy and pathological synovial fluid joints and tendon sheaths (PCA: R^2^X = 0.88, Q2 = 0.684; OPLS-DA: R^2^X = 0.775; R^2^Y = 0.6772, Q2 = −0.432). Multivariate statistical analysis revealed distinct clustering of healthy joints samples grouping closely with pathological joints samples (OPLS-DA: R^2^X = 0.662; R^2^X = 0.859, Q2 = 0.786). These findings were supported by univariate analysis (t-test, p < 0.05). Similarly, multivariate statistical analysis showed strong discrimination between healthy and pathological tendon sheaths synovial fluid (OPLS-DA: R^2^X = 0.742; R^2^Y = 0.892, Q2 = 0.842), also supported by univariate analysis (t-test, *p* < 0.05).

**Discussion:**

Metabolomic profiling by ^1^H-NMR effectively distinguished healthy from pathological synovial fluid in joints and tendon sheaths, providing a clear metabolic fingerprint of disease-related alterations that may support earlier detection and a better understanding of equine musculoskeletal disorders. The main limitation of this study was the small sample size, particularly for tendon sheath samples. Additional synovial fluid specimens from both healthy and pathological joints and tendon sheaths would be needed to implement metabolomic data. High-resolution ^1^H Nuclear Magnetic Resonance spectroscopy proves to be a valuable tool for differentiating healthy from pathological equine synovial fluid. Metabolomic analysis revealed a specific metabolic fingerprint in diseased joints and tendon sheaths, supporting its potential role in the diagnosis and monitoring of orthopedic conditions in horses.

## 1 Introduction

Joints are considered complex organs closely related to the musculoskeletal system, which is essential for movement and athletic performance. Any traumatic injury affecting the joint organ can damage the articular cartilage, leading to its degradation and, eventually, to chronic joint disorders such as osteoarthritis (OA) (1).

OA is the most common disease affecting joints and is a major cause of pain, disability, and economic loss in humans worldwide (2). Joint diseases, including OA, are similarly relevant in equine species (3), as they are a leading cause of lameness, reduced performance, and, consequently, early retirement from competition in athletic horses (4).

Lameness in horses frequently involves synovial joints, characterized by the presence of synovial fluid (SF), which plays a key role in joint homeostasis through the balance of anabolic and catabolic metabolic processes (5).

Repeated trauma in athletic horses may induce synovitis and capsulitis (6), with the release of inflammatory mediators that disrupt this balance and contribute to degenerative changes (7).

To provide an earlier and more accurate diagnosis of OA, a technique based on the detection of biomarkers from the tissues undergoing metabolic changes in the early stages of OA has been investigated in both human (8–10) and veterinary medicine (11–13).

Biomarkers are molecules produced physiologically during metabolic processes. During inflammation, alterations in the balance between metabolic pathways cause qualitative and quantitative changes in biomarkers composition. Therefore, the identification and quantification of biomarkers in synovial fluid, serum, and urine offer an opportunity to employ them as indicators of joint diseases (5).

Biomarker analysis has the advantage of being a simple, repeatable, and minimally invasive procedure (14). Potential applications of biomarkers include the acquisition of a chemical ‘fingerprint’ of specific cellular processes that can be correlated with various physiological or pathological conditions (15, 16), supporting the early investigation of OA-related changes in joints, the differentiation between affected and unaffected joints, the assessment of articular cartilage degradation, and the monitoring of therapeutic response (5).

In human medicine, numerous biomarkers associated with joint disease have been identified and studied for their potential diagnostic and prognostic value. However, despite significant progress, none has yet been universally validated for reliable early diagnosis of OA in clinical practice (17). Similarly, in veterinary medicine, research on biomarkers as indicators of joint pathology and as tools for objectively assessing therapeutic efficacy is rapidly evolving but still largely exploratory, with few long-term or large-scale studies in animals affected by naturally occurring disease.

In addition to biomarkers in joints affected by OA, biomarkers in joints of horses with osteochondritis dissecans (OCD) are also described (18).

In horses affected by OCD, the detection of biomarkers produced by the metabolism of cartilage and bone within synovial fluid may provide important data on joint condition and eventually on prognosis (18).

Furthermore, another significant cause of lameness in performance horses and economic loss due to early career-ending is tendon injuries (19).

Similar to OA and OCD, the study of biomarkers in SF from tendon sheaths affected by tendon injuries provides useful information for the early diagnosis of tenosynovitis and for assessing the effectiveness of treatment and rehabilitation. Markers that have been studied intensively include carboxyterminal propeptide of type I collagen (PICP), cross-linked carboxyterminal telopeptide of type I collagen (ICTP) (20), and cartilage oligomeric matrix protein (COMP) (21–23).

This study aims to perform a metabolite analysis to investigate the biomarkers within the synovial fluid of normal and pathological joints and normal and pathological tendon sheaths of horses. The metabolite analysis was performed with the high-resolution ^1^H Nuclear Magnetic Resonance (^1^H-NMR). Although the detection of biomarkers can be performed on different matrices such as serum, plasma, blood, and urine (24), the degradation products, enzymes, and signal transduction molecules involved in any orthopedic disease are mainly released in SF from the surrounding joint tissue, providing valuable biochemical information on the metabolic status of the affected joint (24–26).

^1^H-NMR enables the evaluation of several low molecular-weight components, which can be observed and quantified simultaneously with a margin of error of ± 5% (27). Among the analytical techniques available, ^1^H-NMR is a widely used in human medicine for orthopedic diseases, e.g., to distinguish patients with diffuse-type tenosynovial giant cell tumor from healthy control (28), to investigate synovial fluid from patients with osteoarthritis (16) and rheumatoid arthritis (29, 30), and to identify potential biomarkers and perturbed metabolic pathways in osteoarthritis (31).

Additionally, in veterinary medicine, the application of the ^1^H-NMR is reported, especially in dogs, e.g., to study synovial fluids from patients affected by spontaneous osteoarthritis (32), to determine metabolomic biomarkers of meniscal injury in dogs with cranial cruciate ligament rupture (33), or to monitor the effectiveness of therapy in dogs affected by osteoarthritis (34).

In horses, ^1^H-NMR has been applied to the study of synovial fluid metabolomics to differentiate healthy joints from those affected by OA (35–37). However, studies investigating biomarkers in healthy and pathological tendon sheaths are currently lacking.

We also suppose that the ^1^H-NMR-based metabolomic analysis is a valuable tool for analyzing the metabolite profile of normal and pathological joints and normal and pathological tendon sheaths of horses to facilitate an early diagnosis of joint and tendon injuries and to develop an objective method of evaluating the effectiveness of therapies.

## 2 Materials and methods

### 2.1 Study design

Prospective clinical study.

### 2.2 Animal welfare

A written informed consent form was prepared and provided to the owner, who, after being fully informed about the study, signed it and agreed to the animal’s inclusion.

The study was conducted after the approval of the Ethical Committee of Veterinary Clinical and Zootechnical Studies of the Department of Precision and Regenerative Medicine and the Ionian area of the University of Bari (approval number: 2873–1 III/13 on 30/04/2025).

### 2.3 Population and experimental groups

For the purpose of the study, SF from healthy joints and tendon sheaths from regularly slaughtered animals, and SF from pathologic joints and tendon sheaths from owned athlete horses screened were employed at the Surgical Unit of the Section of Veterinary Clinics and Animal Production of the Department of Precision and Regenerative medicine and the Ionian area (DiMePre-J) of the University of Bari and at the Faculty of Veterinary Medicine of Leipzig. The collected samples of SF were assigned to the following experimental groups: healthy joints (H-J), pathologic joints (P-J), healthy tendon sheaths (H-TS), and pathologic tendon sheaths (P-TS).

### 2.4 Sample size

Given the exploratory nature of this study and the lack of prior metabolomic data for equine tendon sheaths, no formal sample size calculation was performed *a priori*. Instead, the sample size was determined based on the availability of eligible clinical cases and healthy specimens, in line with previously published ^1^H-NMR metabolomic studies in veterinary medicine (35, 36). Nevertheless, to assess the adequacy of the collected dataset, a *post hoc* power analysis was performed using G*Power 3.1 software. The analysis was based on univariate comparisons of key discriminant metabolites identified through multivariate models. Assuming an alpha level of 0.05 and an effect size (Cohen’s d) of 1.0, which corresponds to the observed magnitude of differences in normalized spectral intensities, the calculated power exceeded 80% for most group comparisons. These findings suggest that, despite the relatively limited number of tendon sheath samples, the study was sufficiently powered to detect biologically relevant differences in metabolic profiles between healthy and pathological synovial fluids.

### 2.5 Inclusion and exclusion criteria

For the H-J and H-TS groups, synovial fluids were collected from regularly slaughtered horses, less than 2 years old. Before collecting the samples, all animals were assessed. Only lameness-free horses with good general conditions, no signs of systemic disease, and no visible injuries to the limbs (e.g., wounds, swelling, exudations, etc.) were included for SF sampling. For the purpose of the study, samples of SF were collected from healthy metacarpal-phalangeal joints (H-J group) and flexor tendon sheaths (H-TS group) of forelimbs.

Regarding the P-J and P-TS groups, all horses presented at the clinic for lameness were examined. The evaluation of body condition score (BCS) based on the Henneke 1–9 scoring system (38), a full orthopedic, radiographic, and ultrasonographic examinations were performed. For the sample collection, horses with a diagnosis of OA and OCD involving distal interphalangeal, metacarpal/metatarsal-phalangeal, carpal, talocrural, tibiotarsal, or femorotibial joints and synovitis of the flexor tendon sheath were enrolled. The recruited horses showed lameness at least equal to or above 1/5 (according to the American Association of Equine Practitioners 5-point scale), positive flexion test, and positive intra-articular anesthesia. The radiological diagnosis of OA or OCD was realized in orthogonal standard and oblique projections. X-ray examinations were evaluated by two radiologists and classified by assessing the degree of osteophytosis according to a previously published score (39). In addition, an ultrasound examination was performed by the same operator to diagnose bursitis, synovitis, injury, and other tendinopathies of the tendon sheaths. Moreover, subjects undergoing anti-inflammatory therapy with NSAIDs or intra-articular treatment with steroid drugs and/or with hyaluronic acid within 3 weeks were not included. Furthermore, horses with acute injuries with open and infected wounds (septic arthritis or septic tenosynovitis) were excluded.

### 2.6 Collection of synovial fluid and sample preparation for ^1^H-NMR

Synovial fluid samples were aliquoted on ice immediately after collection. Samples scheduled for analysis within 12 h were stored at −20 °C, while those requiring longer storage were frozen at −80 °C for a maximum of 3 months. All samples were transported and processed under frozen conditions, following identical procedures to minimize freeze–thaw cycles and preserve metabolite stability. This workflow ensured consistent pre-analytical conditions, minimized freeze–thaw cycles, and preserved metabolite stability (40).

The analysis of SF samples was performed at the General and Inorganic Chemistry Laboratory of the Department of Biological and Environmental Sciences and Technologies (DiSTeBA), University of Salento. The samples were prepared as previously reported (35, 41). After defrosting, each sample was spun down in a microcentrifuge at 18,000 rpm for 5 min (temperature 4 °C). Then, 350 μL of each SF sample was added to 350 μL of ultrapure deionized water and 100 μL of phosphate buffer [1 M Na_2_HPO_4_/NaH_2_PO_4_ in D_2_O containing DSS (sodium 4,4-dimethyl-4-silapentane-1-sulfonate) as a chemical shift reference and NaN_3_ 2 mmol/L to prevent bacterial contamination] at pH 7.4 (*δ* = 0 ppm). After further centrifugation at the above-described conditions, 600 μL of the solution was filled in a 5-mm ^1^H-NMR tube.

### 2.7 ^1^H-NMR study

Spectra acquisition was performed at 300 K on a Bruker Advance III 600 Ascend NMR spectrometer (Bruker, Karlsruhe, Germany) operating at 600.13 MHz, equipped with a TCI CryoProbe (inverse triple resonance Cryoprobe Prodigy), a z-axis gradient coil, and automatic tuning-matching (ATM). The samples were placed in an autosampler (BASC-60 autosampler: Burker Automatic Sample Charger) interfaced with IconNMR software, and the acquisition of NMR spectra proceeded automatically. For each sample, a 1D ^1^H one-dimensional CPMG (referred to as Carr–Purcell–Meiboom–Gill spin-echo sequence) experiment with a transverse-relaxation-filter incorporating pulse sequence was run with 128 scans, 16 dummy scans, 64 K time domain, spectral width 20.0276 ppm (12,019.230 Hz), 5 s relaxation delay (d1), 12.380 μs 90 degree high power pulse (p1), and 1.36 s acquisition time (AQ). The resulting Free Induction Decays (FIDs) were multiplied by an exponential weighting function corresponding to a line broadening (LB) of 0.3 Hz before Fourier transformation, automated phasing, and baseline correction. Metabolite identifications were based on ^1^H and ^13^C assignment by 1D and 2D homo- and heteronuclear experiments (2D ^1^H Jres, ^1^H COSY, ^1^H-^13^C HSQC, and HMBC) and by comparison with the literature data (32, 34, 35).

### 2.8 Metabolites panel

- Amino acids

 - 2-Amynobutyrate, 5-aminolevulinate, alanine, asparagine, creatine, creatinine, valine, glutamine, glycine, guanidoacetate, histidine, isoleucine, leucine, lysine, methionine, N-acetyl-amino acid, ornithine, phenylalanine, proline, saronine, valosin, taurine, and tyrosine.

- Fatty and organic acids

 - 𝛽-Hydroxybutyrate, 2-hydroxybutyrate, 3-𝛽hydroxybutyrate, 3-hydroxyisovalerate, acetate, acetoacetate, carnitine, citrate, formate, lactate, malonate, pyruvate, and glycerol.

- Sugars

 - Acetylated saccharide, glucose, mannose, and myoinositol.

- Other

 - Formic acid, acetylcholine, adenosine, choline, dimethylamine, ethanol, isopropanol, mobile-lipid, o-phosphocoline, oxipurinol, sn-glycero-3-phosphocoline, and xanthine.

### 2.9 Statistical analysis

The ^1^H-NMR spectra were processed using Topspin 3.6.4 and Amix 3.9.13 (Bruker, Biospin, Italy), both for simultaneous visual inspection and the successive bucketing process. All NMR spectra (in the range 9.00–0.85 ppm) were segmented into fixed rectangular buckets of 0.04 ppm width (normal rectangular bucketing). To avoid interference, the spectral regions between 5.18 and 4.67 ppm were discarded because of the residual peak of water. Signals assigned to EDTA (3.62, 3.22 ppm) and gluconate (4.14, 4.04, 3.82, 3.78, 3.74, 3.66 ppm) contamination were further excluded from statistical analysis. The total sum normalization was applied to minimize small differences due to sample concentration and/or experimental conditions among samples (42). The data results created a matrix in which we can recognize the bucketed ^1^H-NMR spectra values (in columns) and the corresponding sample (in rows). Then, multivariate statistical analysis [unsupervised principal component analysis (PCA) and the supervised orthogonal partial least squares discriminant analysis (OPLS-DA)] was performed to examine the intrinsic variation in the data, using SIMCA 14 software (Sartorius Stedim Biotech, Umeå, Sweden). The Pareto scaling procedure was applied, performed by dividing the mean-centered data by the square root of the standard deviation. The robustness of the statistical models was tested by cross-validation default method (7-fold) and further evaluated with a permutation test (400 permutations). The quality of the models (the total variations in the data and the internal cross-validation) was described by R^2^ and Q^2^ parameters (43). The relative change in discriminating metabolites, represented as mean intensities and ± standard deviation (SD) between the observed groups, was validated by analyzing the mean values +/− standard deviation of selected buckets. Reduced distinctive unbiased NMR signals were validated using a univariate t-test, using the free “Metaboanalyst “version 5.0 software (44). Statistical significance was set at least at an adjusted *p*-value < 0.05.

## 3 Results

### 3.1 Sample collection

Concerning the healthy groups, 19 horses met the inclusion criteria. From these horses, 12 samples of SF were collected from the metacarpal-phalangeal joint, and 19 samples of SF from the flexor tendon sheaths of forelimbs. Data recorded for the enrolled horses include sex (9 females and 10 stallions), age (mean 2 years ± SD), and BCS (mean 6 ± SD). Whereas, for pathologic groups, 31 mixed-breed performance horses were included in the study. Data recorded for enrolled horses include sex (13 females, 4 stallions, 14 geldings), age (mean 7 years ± SD), and BCS (mean 5 ± SD). Specifically, 18 samples of SF from joints affected by OCD/OA (5 samples from stifle joints, 5 samples from talocrural joints, 4 samples from fetlock joints, 1 sample from pastern joint, and 3 samples from coffin joints) and 6 samples from pathological flexor tendon sheaths (1 with bursitis, 2 with traumatic synovitis, 2 with non-specific injuries, and 1 with tendinopathy of digital flexor tendon) were collected. All data concerning samples of SF are summarized in Tables 1, 2.

**Table 1:** Samples of SF collected for pathological joints (P-J group).

Parameter	Stifle joints	Talocrural joints	Fetlock joints	Pastern joints	Coffin joints
Number of samples	5	5	4	1	3
Forelimb (F)/Hindlimb (H)	3 F + 2 H	3 F + 2 H	3 F + 1 H	H	2 F + 1 H
Left (L)/Right (R)	1 L + 4 R	3 L + 2 R	3 L + 1 R	R	2 L + 1 R
Diagnosis (OA/OCD)	OA	2 OA + 3 OCD	2 OA + 2 OCD	OA	OCD

Distribution of synovial fluid samples collected from joints affected by OA and OCD. The table includes the number of samples, anatomical localization, and diagnosis.

**Table 2:** Samples of SF collected for pathological tendon sheaths (P-TS group).

Parameter	Flexor tendon sheaths			
Number of samples	1	2	2	1
Forelimb (F)/Hindlimb (H)	F	F	F	H
Left (L)/Right (R)	L	1 L + 1 R	1 L + 1 R	R
Diagnosis	Bursitis	Injuries	Synovitis	Tendinopathy

Distribution of synovial fluid samples collected from pathological tendon sheaths. The table includes the number of samples, anatomical localization, and diagnosis.

### 3.2 ^1^H-NMR metabolomic analysis

**Figure 1:**
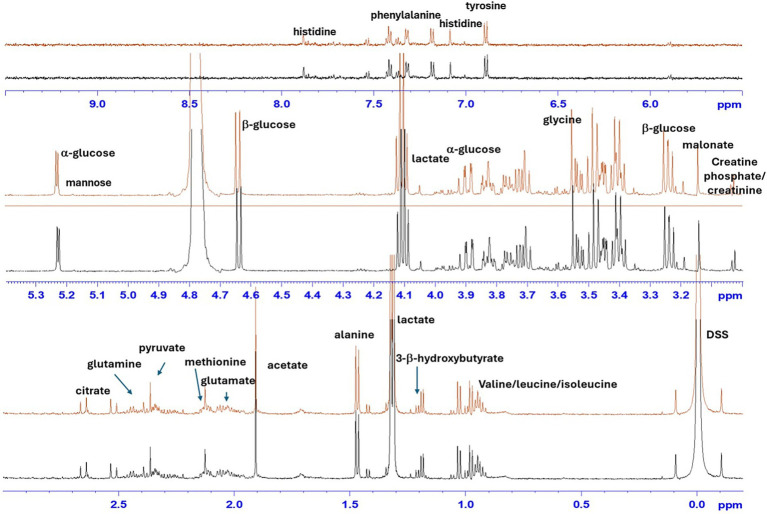
Stacked plot of ^1^H-NMR spectra of representative SF sample collected from healthy joints (H-J) and tendon sheaths (H-TS). Assigned metabolites are indicated.

The ^1^H-NMR metabolomic analysis revealed the presence of different metabolites in the analyzed samples. In particular, as observed by the reported stacked plot of synovial fluid from healthy joints (H-J) and tendon sheaths (H-TS) (Figure 1), the main assigned metabolites are amino acids (valine, leucine, isoleucine, alanine, glycine, tyrosine, and phenylalanine), organic acids (lactate, citrate, and pyruvate), sugars (*α*/*β* glucose), and other metabolites (such as creatine/creatinine, malonate, and 3-β-hydroxybutyrate).

To analyze the potential differences in the metabolic profile of the considered samples, NMR data were then submitted for chemometric analysis.

**Figure 2:**
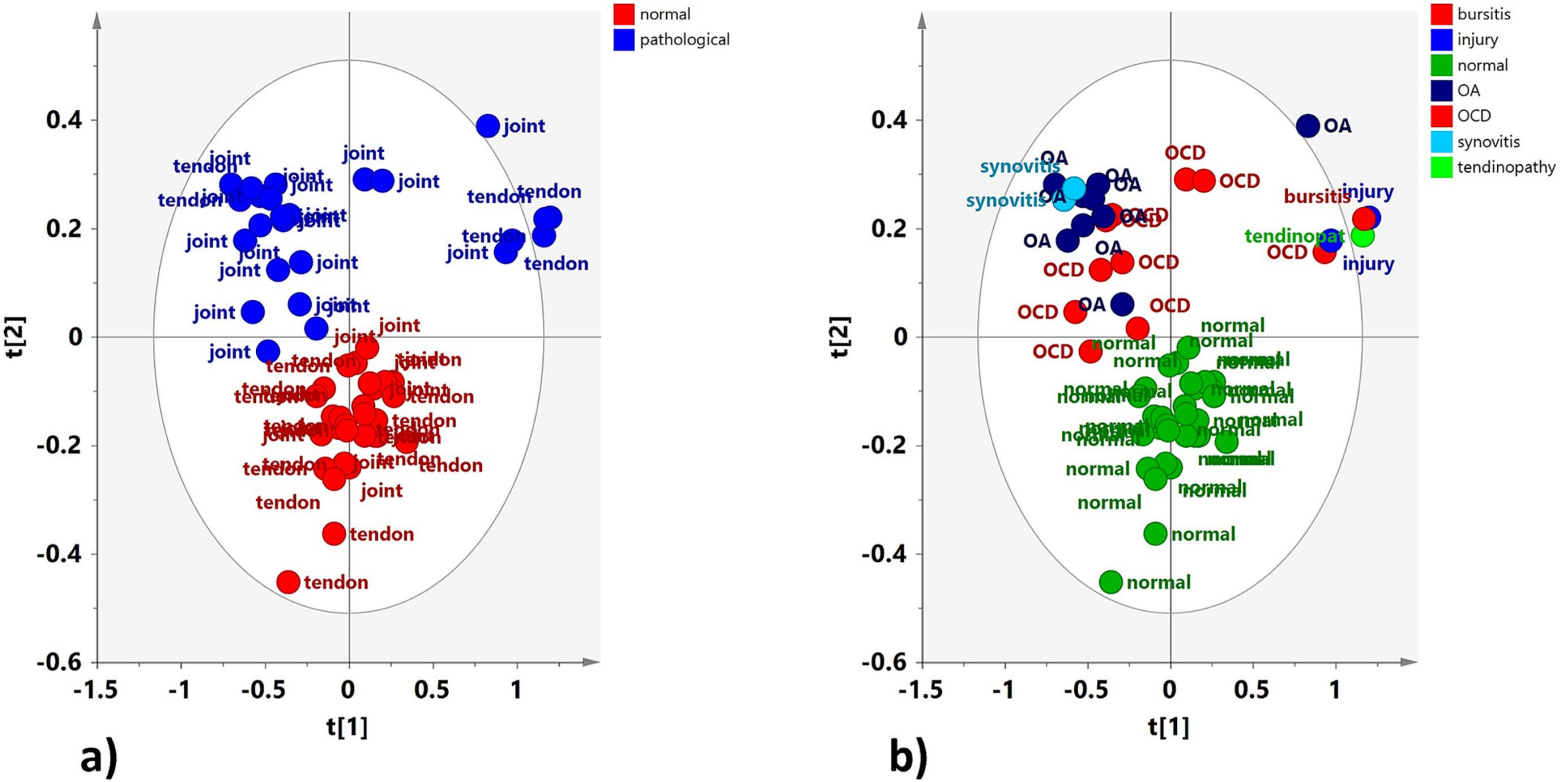
PCA (5 components, R^2^X = 0.817; Q^2^ = 0.657) scores plot for the whole normal (H-J, H-TS) and pathological (P-J, P-TS) joints and tendon sheaths samples, respectively. Observations in the figures are colored according to **(a)** equine status and matrix type (joints and tendon sheaths) and **(b)** diagnosis.

The preliminary unsupervised analysis of the whole dataset (H-J, H-TS, P-J, and P-TS samples) showed a clear clustering of the observations along the second component (t2). Interestingly, the P-TS group grouped with two P-J samples, forming a subcluster at positive t1/t2 values in the scores plot (Figure 2).

Explorative PCA was then applied separately to the H-J and the H-TS groups, and to the P-J and the P-TS groups.

**Figure 3:**
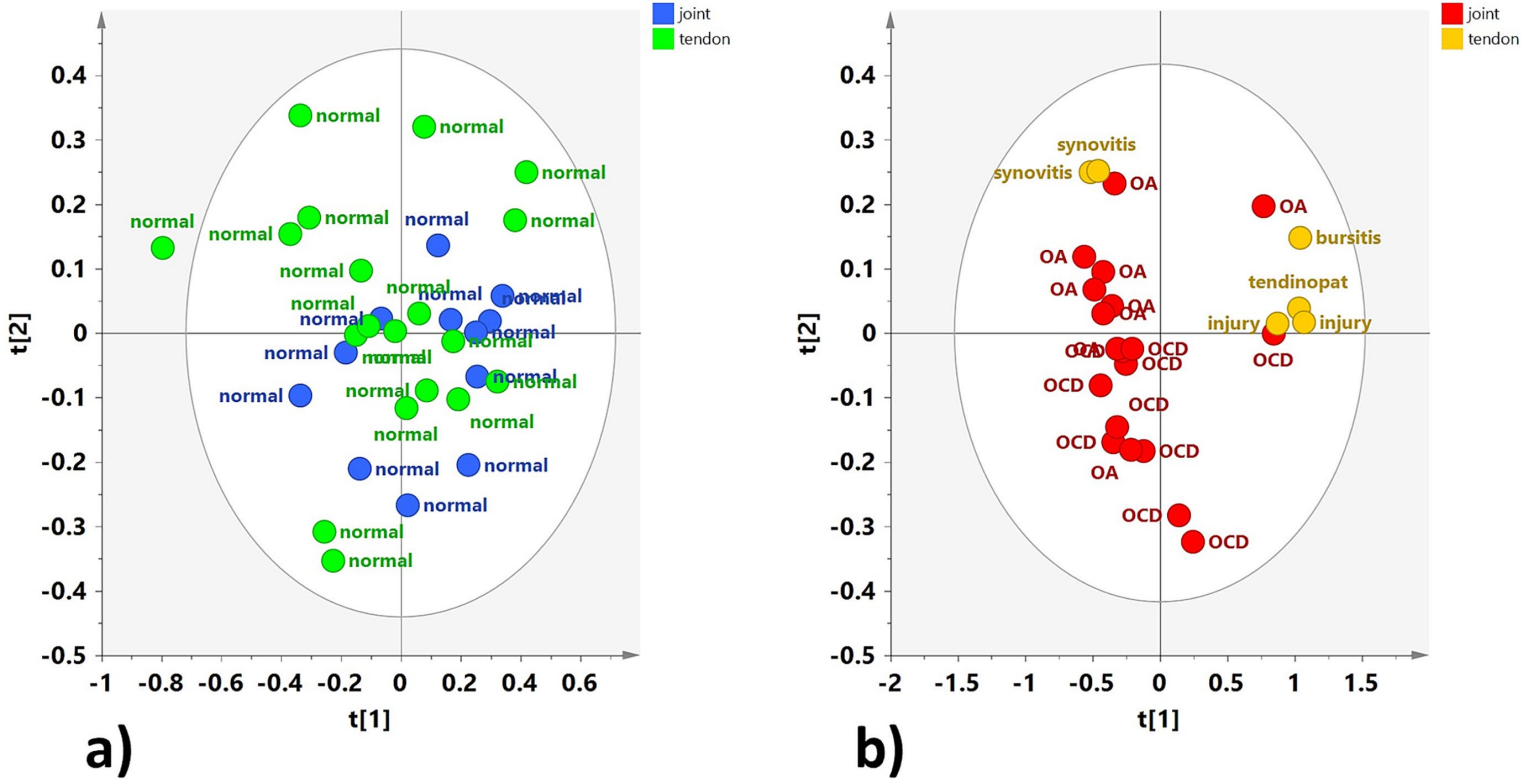
PCA scores plot for whole **(a)** normal joints (H-J) and tendon sheaths (H-TS) (5 components, R^2^X = 0.761, Q^2^ = 0.372), and **(b)** pathological joints (P-J) and tendon sheaths (P-TS) samples (3 components, R^2^X = 0.88, Q^2^ = 0.684).

No specific clustering was observed in the healthy dataset, suggesting a similar metabolite composition between the H-J and the H-TS groups (Figure 3a). On the contrary, despite the limited number of tendon sheath SF samples, a clear separation between the P-J and the P-TS groups was observed, except for two samples of the P-TS group with the diagnosis of synovitis, placed close to the P-J group samples (Figure 3b).

**Figure 4:**
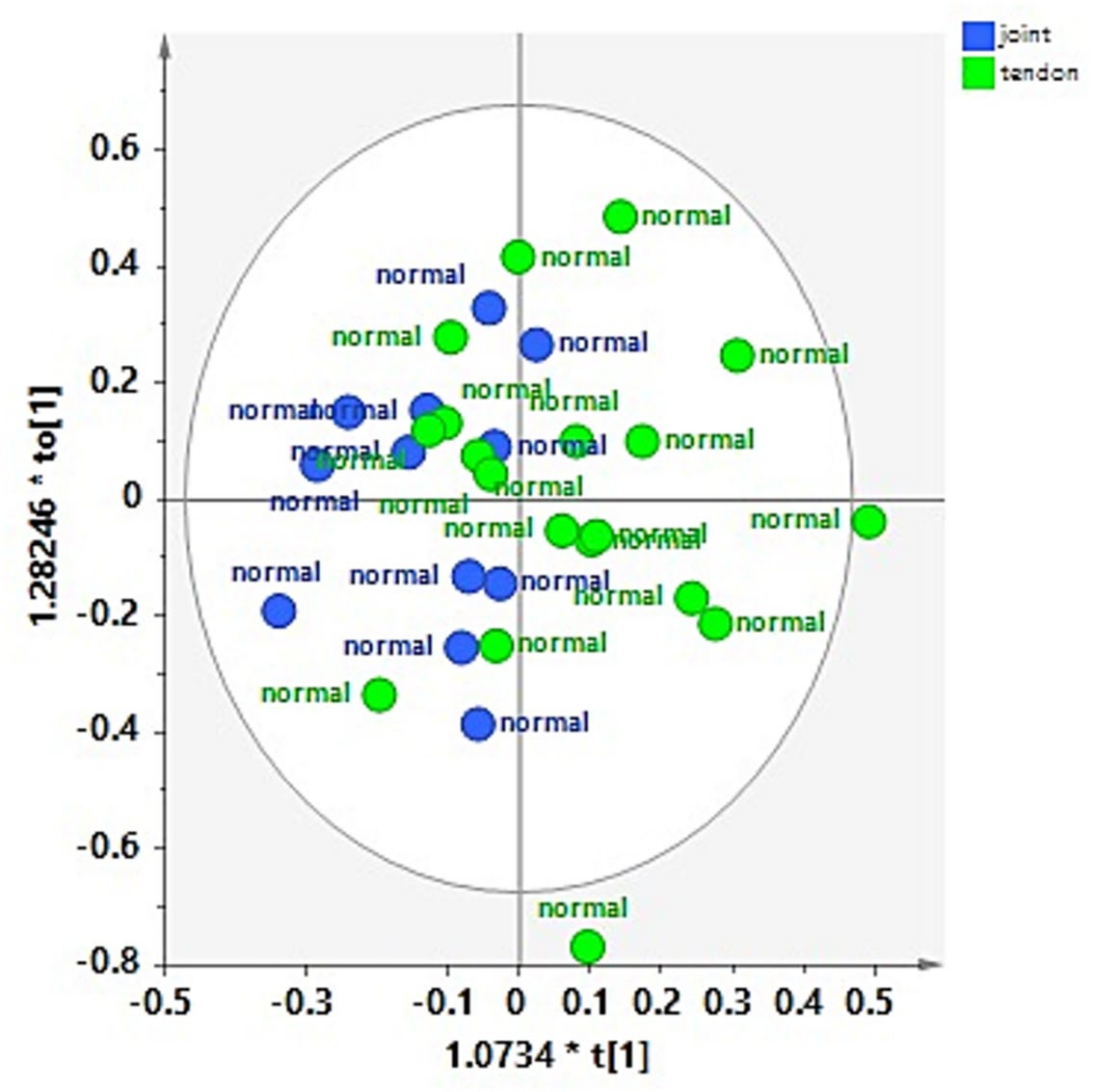
OPLS-DA scores plot for the normal joint (H-J) and tendon sheath (H-TS) class samples (1 + 1 + 0, R^2^X = 0.48; R^2^Y = 0.292, Q^2^ = −0.143).

These observations were refined and clearly observed in the OPLS-DA pairwise comparison and relative descriptive and predictive parameters (R^2^X, R^2^Y, and Q^2^). Thus, the OPLS-DA pairwise of the H-J and the H-TS groups confirmed that no specific separation was observed between the considered classes, exhibiting a negative predictive parameter (Q^2^ = -0.143) (Figure 4).

**Figure 5:**
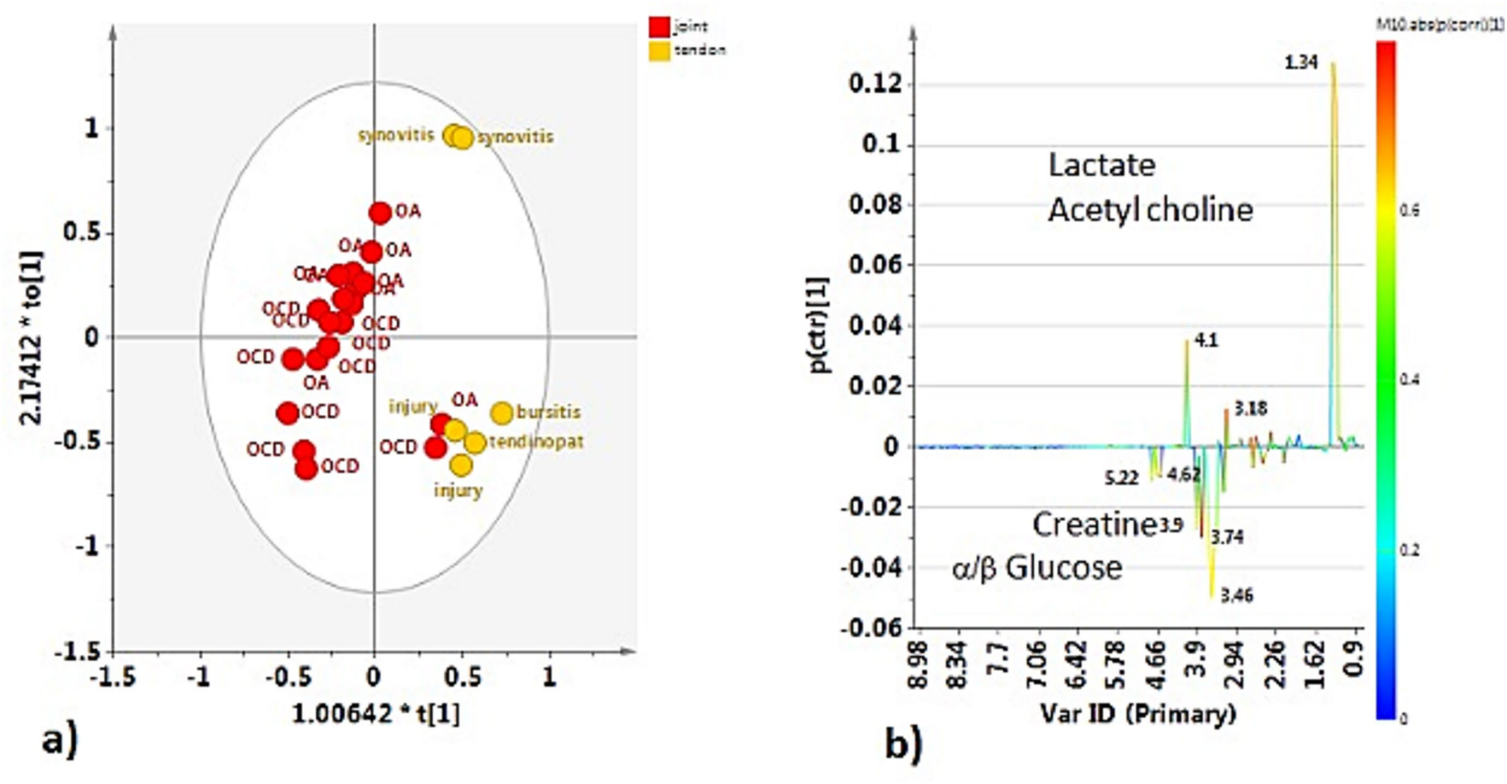
**(a)** OPLS-DA scores plot for the pathological joint (P-J) and tendon sheath (P-TS) class samples (1 + 1 + 0, R^2^X = 0.775; R^2^Y = 0.6772, Q^2^ = −0.432). **(b)** S-line plot for the model colored according to the correlation scaled coefficient (p-corr), displayed for the first component.

The OPLS-DA comparing the P-J and P-TS classes confirmed the separation between pathological joints and tendon sheaths SF samples, except for two P-J samples (Figure 5a). The descriptive and predictive parameters of the model were quite good, indicating differences in the metabolic profiles of the two examined matrices. In fact, the S line plot for the model showed as the P-TS class exhibited a higher content of lactate (bins at 1.34 and 4.1 ppm) and acetylcholine (bin at 3.18 ppm); whereas, the P-J class is characterized by a higher content of *α*/*β* glucose (bins at 5.22 ppm and 4.62 ppm for α and β, respectively) and creatine (bin at 3.90 ppm) (Figure 5b).

**Figure 6:**
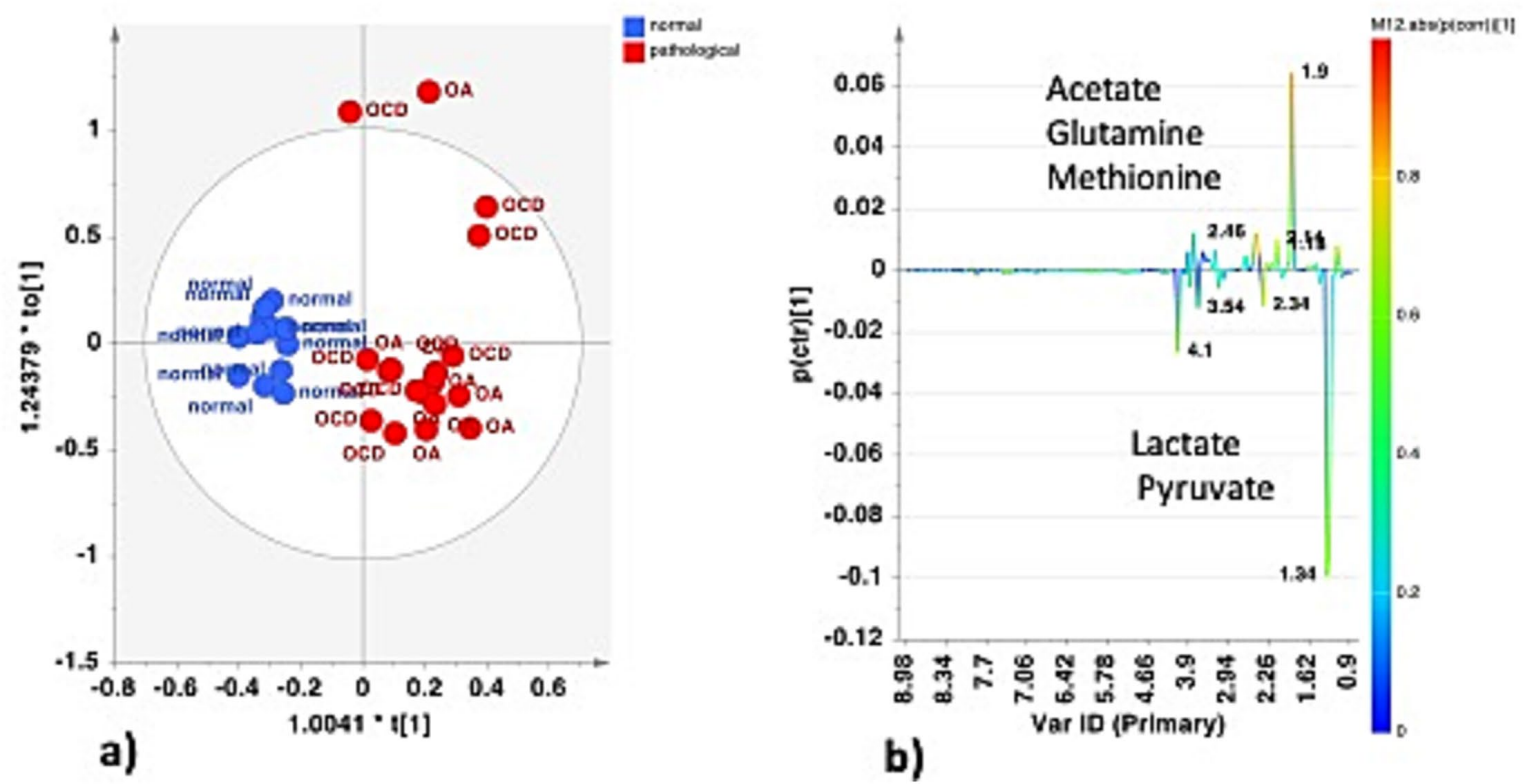
**(a)** OPLS-DA scores plot for the normal (H-J) vs. pathological (P-J) joint class samples (1 + 1 + 0, R^2^X = 0.662; R^2^Y = 0.859, Q^2^ = 0.786). **(b)** S-line plot for the model colored according to the correlation scaled coefficient (p-corr), displayed for the first component.

Joints and tendon sheaths SF equine samples were then studied separately to focus on the possible differences in the metabolic profiles between normal and pathological matrices. In the case of joint samples, after explorative PCA (data not shown), supervised OPLS-DA analysis was performed (Figure 6a). The strong statistical parameters of the model revealed a clear partition among the class samples, characterized by a higher content of lactate (bins at 1.34 and 4.1 ppm) and pyruvate (bin at 2.34 ppm) for the H-J class and a higher continent of acetate (bin at 1.90 ppm), glutamine (bin at 2.46 ppm), and methionine (bin at 2.14 ppm) for the P-J class (Figure 6b).

**Figure 7:**
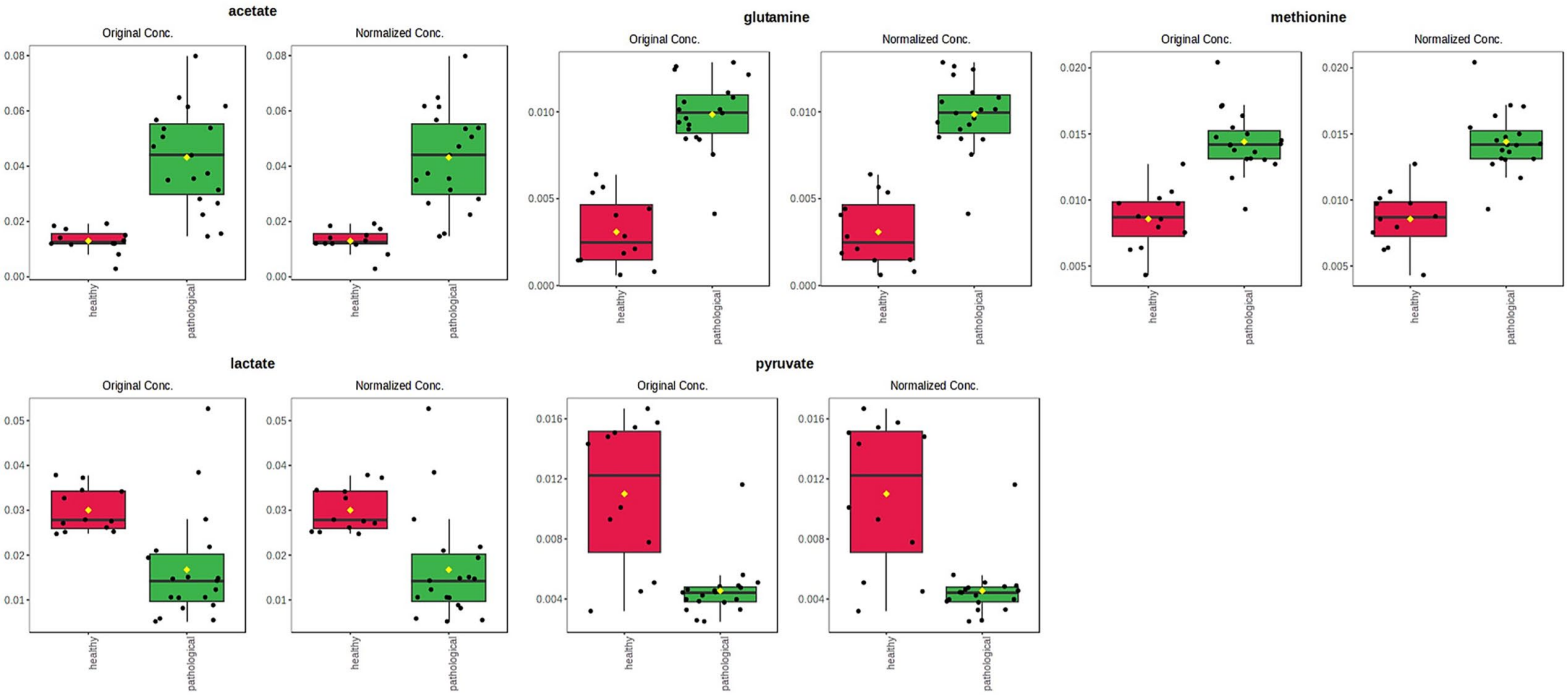
Box and whisker plots of significantly (t-test; *p* < 0.05) discriminant metabolites (assigned binned peak) identified from OPLS-DA analysis. Y axes are represented as relative units. Data were mean-centered and normalized to the total spectral area. The bar plots on the left show the original values (mean ± SD). The box and whisker plots on the right summarize the normalized values. Notch indicates the 95% confidence interval for the median; whiskers exclude outliers. The mean value of each group is indicated as a yellow diamond.

Univariate analysis (two samples, t-test) confirmed a statistically significant difference in discriminating metabolites (assigned binned peak) as represented on their associated box plots (Figure 7). Specifically, acetate (bin at 1.90 ppm, *p* = 3.7 × 10^−6^), glutamine (bin at 2.46 ppm, *p* = 7.7 × 10^−10^), and methionine (bin at 2.12 ppm, *p* = 1.6 × 10^−7^) were significantly higher in the P-J group, whereas lactate (bin at 1.34 ppm, *p* = 0.0011) and pyruvate (bin at 2.36 ppm, *p* = 1.6 × 10^−5^) were significantly higher in the H-J group (Supplementary Table S1).

**Figure 8:**
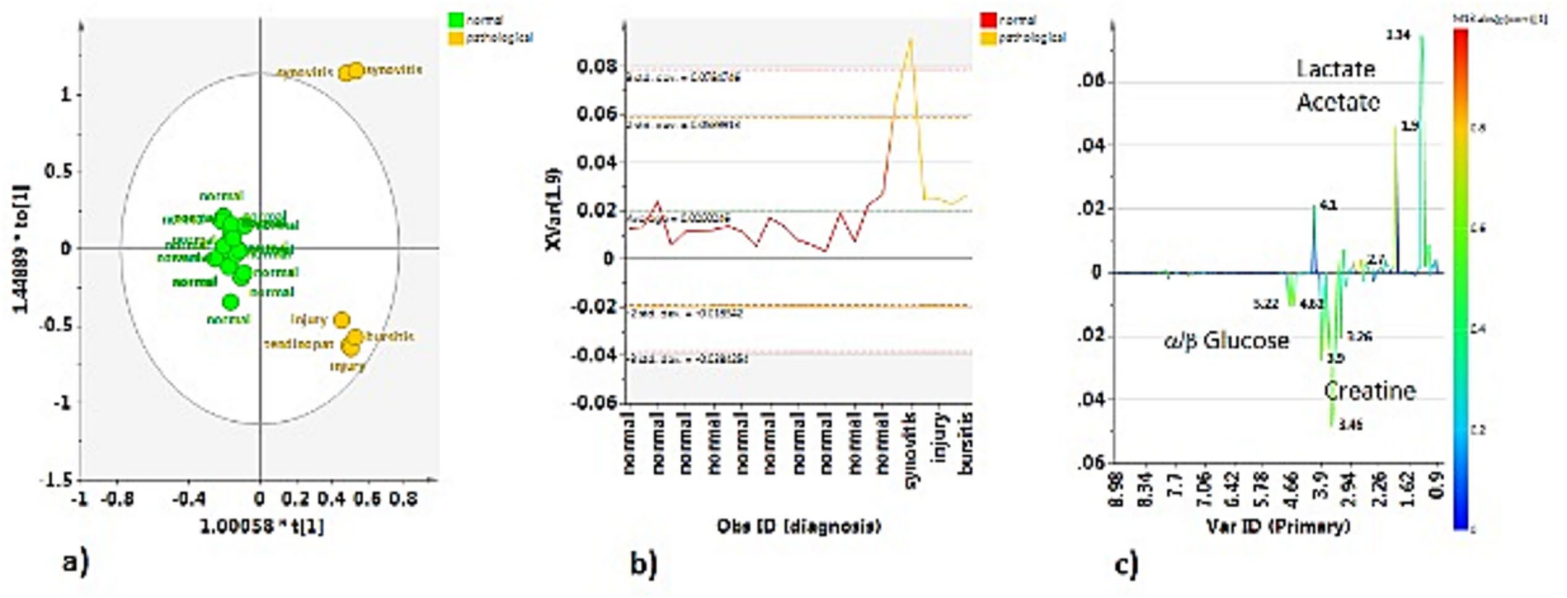
**(a)** OPLS-DA scores plot for the normal (H-TS) vs. the pathological (P-TS) tendon sheath class samples (1 + 1 + 0, R^2^X = 0.742; R^2^Y = 0.892, Q^2^ = 0.842). **(b)** Variable trend plot with control limits of selected discriminating X variable (1.9 ppm). **(c)** S-line plot for the model colored according to the correlation scaled coefficient (p-corr), displayed for the first component.

Comparison between the H-TS and P-TS classes, after a preliminary unsupervised PCA (data not shown), reveals, also in this case, a good separation between the considered classes, also indicated by the excellent statistical parameters for the model (Figure 8a).

The two P-TS class samples with the diagnosis of synovitis, placed together, dispersed along the first orthogonal component, characterized by a higher content of acetate (bin at 1.90 ppm), as observed from the relative variable trend plot (Figure 8b). The S line for the model is revealed in this case as the P-TS class is characterized by elevated lactate (bins at 1.34 and 4.1 ppm) and acetate (bin at 1.90 ppm), whereas the H-TS class exhibited *α*/*β* glucose (bins at 5.22 and 4.62 for α and β, respectively) and creatine (bin at 3.90 ppm) as discriminant compound (Figure 8c).

**Figure 9:**
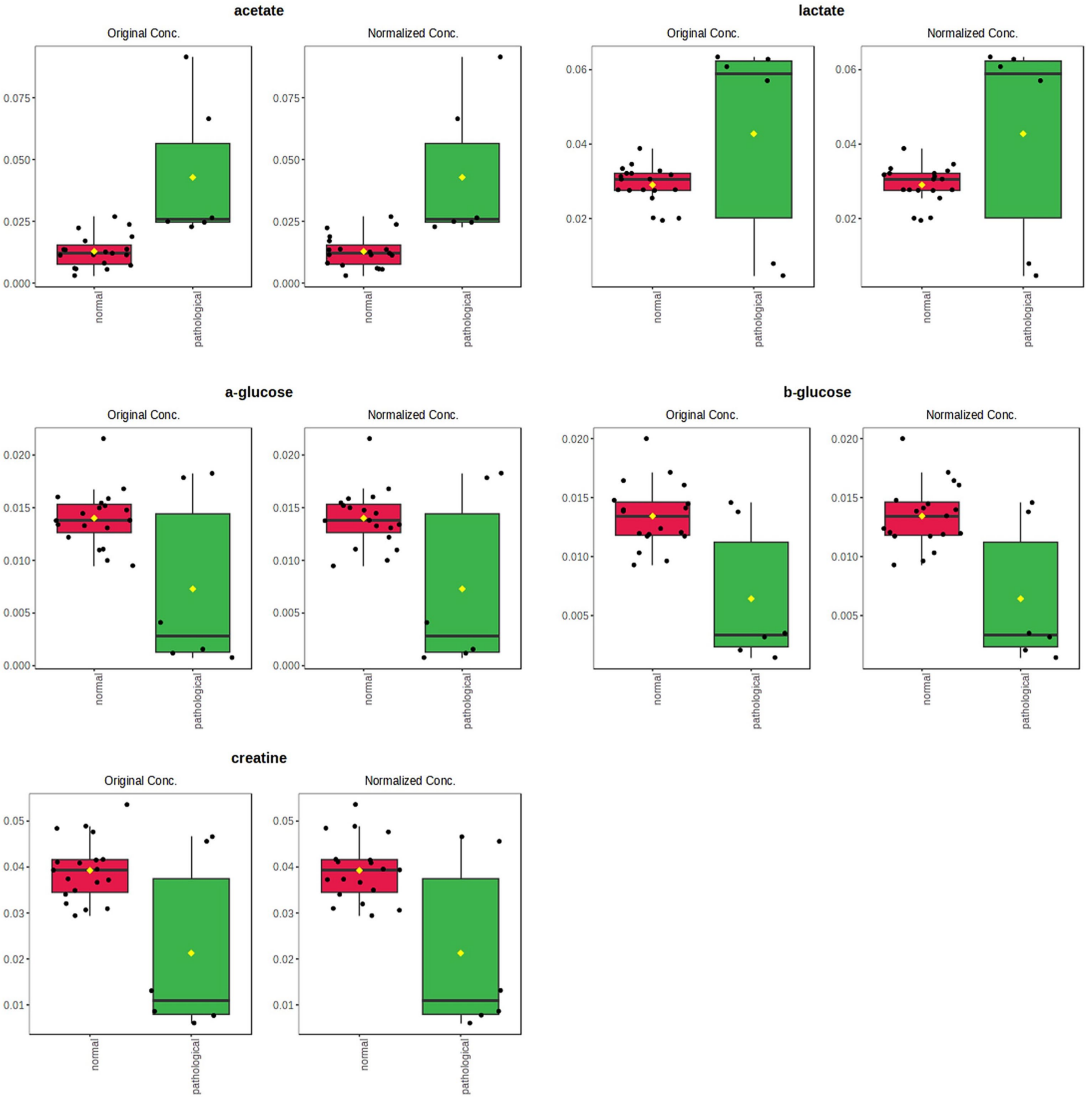
Box and whisker plots of significantly (t-test; *p* < 0.05) discriminant metabolites (spectral bins) identified from OPLS-DA analysis. Y-axes are represented in relative units. Data were mean-centered and normalized to the total spectral area. The bar plots on the left show the original values (mean ± SD). The box and whisker plots on the right summarize the normalized values. Notch indicates the 95% confidence interval for the median; whiskers exclude outliers. The mean value of each group is indicated as a yellow diamond.

Discriminating metabolites (binned signals) were then validated by univariate analysis to validate the statistical significance in the differentiation, as reported in the relative box plots (Figure 9). Specifically, acetate (bin at 1.90 ppm, *p* = 0.0471) and lactate (bin at 1.34 ppm, *p* = 7.66 × 10^−10^) were significantly higher in the P-TS group, whereas α-glucose (bin at 5.22 ppm, *p* = 0.0050) and β-glucose (bin at 4.62 ppm, *p* = 5.06 × 10^−4^) were significantly higher in the H-TS group (Supplementary Table S2).

## 4 Discussion

Metabolomic analysis performed in this study showed characteristic spectra of synovial fluid metabolites from healthy and pathological joints (the H-J and P-J classes) and healthy and pathological tendon sheaths (the H-TS and P-TS classes). In particular, overlapping metabolite panels were observed between the H-J and the H-TS classes, whereas distinct metabolite profiles were detected between the P-J and the P-TS classes (except for two P-TS samples diagnosed with synovitis, which exhibited a metabolic profile overlapping with the P-J group), as well as between the H-J and the P-J classes and between the H-TS and the P-TS classes.

The main metabolites identified in the metabolic panels of the H-J and the H-TS classes included amino acids (valine, leucine, isoleucine, alanine, glycine, tyrosine, and phenylalanine), organic acids (lactate, citrate, and pyruvate), sugars (*α*- and *β*-glucose), and other compounds such as creatine/creatinine, malonate, and 3-β-hydroxybutyrate.

The main part of synovial fluid derives from plasma ultrafiltration, enriched with macromolecules locally produced, such as hyaluronic acid. In this way, the synovial membrane of joints and tendon sheaths acts like a filter, allowing the passage of molecules based primarily on their molecular weight (45). In our healthy samples, the detection of free amino acids indicates normal protein turnover and metabolic activity within the joint and tendon sheath environment. Amino acids such as glycine, alanine, and valine are precursors of collagen and elastin and participate in the physiological remodeling of connective tissues (46). Moreover, they participate in cellular metabolism such as the tricarboxylic acid cycle (TCA) cycle and gluconeogenesis to obtain energy (36, 45, 47).

Based on literature, in normal conditions, the joint and the tendon sheath metabolic environment is predominantly aerobic and relies on glycolysis, demonstrated by high levels of pyruvate visible in the synovial fluid of healthy joints spectrum, and *α* and *β* glucose visible in the synovial fluid of tendon sheaths spectrum (48). However, lactate is still produced from glucose, even inside an aerobic environment, together with fatty acid metabolism. This phenomenon, actually called by researchers “paradox of aerobic glycolysis” (49) and peculiar to cartilage and bone, supports the high level of lactate found in the synovial fluid of healthy joints. This mechanism was explained by observing in a rat model, and later in humans (50), that fatty acid oxidation is necessary to supply the right amount of Acetyl Coenzyme A for the TCA cycle; at the same time, it inhibits pyruvate dehydrogenase and, consequently, the conversion of pyruvate into Acetyl Coenzyme A. The pyruvate accumulated is then converted into lactate by the pyruvate-reductase and LDH enzyme, useful to activate the pentose-shunt pathway (45, 49, 50).

Pathological SF samples showed clear group-specific metabolic patterns. The P-J class was characterized by higher *α*/*β*-glucose and creatine concentrations, while the P-TS class exhibited elevated levels of lactate and acetylcholine. Notably, two P-TS samples diagnosed with synovitis displayed a metabolic profile similar to that of P-J samples, suggesting that synovitis may share certain metabolic traits with OA and OCD.

Comparing the H-J class and the P-J class, we obtained important findings regarding discriminating metabolites that characterize the health and disease condition of joints. Under normal physiological conditions, articular cartilage and synovial tissues exhibit a predominantly glycolytic metabolic profile, reflecting their avascular structure and the limited oxygen tension within the joint microenvironment. This metabolic configuration ensures continuous ATP generation and supports biosynthetic activity, enabling cells to adapt rapidly to fluctuations in oxygen availability (48, 51). In this balanced state, lactate and pyruvate act as central intermediates, allowing chondrocytes and synoviocytes to maintain equilibrium between glycolytic and oxidative pathways, thereby preserving redox homeostasis and extracellular matrix turnover (52). Conversely, in osteoarthritic joints, metabolic alterations become evident. The reduction of pyruvate concentrations that we have observed in the P-J group suggests impaired mitochondrial oxidative capacity and a shift toward anaerobic glycolysis, leading to oxidative stress; these metabolic signatures are consistently associated with joint degeneration (53). Moreover, synovial lactate was significantly decreased in the P-J group compared with the H-J group. According to our results, a recent study in dogs with spontaneous OA demonstrated that, alongside higher synovial pH and glucose, lactate concentration was lower in OA joints than in normal joints, supporting a shift away from classical glycolytic flux in chronic disease (54). However, a recent research in which a ^1^H-NMR analysis was conducted on 60 joints demonstrated that lactate levels increased with disease severity, being significantly higher in severe OA than in mild or moderate forms, consistent with a shift toward glycolytic metabolism in affected joints (32). Similarly, a recent equine investigation found that joints affected by osteoarthritis exhibited higher lactate alongside other glycolytic intermediates compared to healthy controls (35). Earlier work in the horse model also reported increased lactate in OA synovial fluid relative to normals (36). Finally, lactate and pyruvate represent promising yet context-dependent metabolic biomarkers of joint pathology, whose diagnostic and prognostic values likely reside in their reciprocal ratio and integration within broader metabolomic profiles rather than in absolute concentration changes alone.

In addition to the statistically significant variation in lactate and pyruvate concentrations, other metabolites such as acetate, glutamine, and methionine also displayed a significant difference pattern between the H-J and the P-J classes. In particular, we observed that acetate, glutamine, and methionine were characterized by an increasing trend in the case of joint disease. Acetate is derived from articular cartilage and synovial fluid breakdown (36). Its increase in joints with osteoarthritis and osteochondritis diseases could be related to the hypoxic and acidotic microenvironment that characterizes the pathological joint (55). Furthermore, this result highlights how fatty acid metabolism plays a predominant role in supplying energy to pathological joints (45). In our study, contrary to previous studies, we recorded low levels of methionine in healthy joints, so in our opinion, the use of methionine as a biomarker of pathology should be clarified (35). Glutamine is an intermediate product of the TCA cycle and is required for glycosaminoglycans (GAG) production; in agreement with the published literature, the finding of elevated glutamine levels can be considered an indicator of low GAG production and thus an indicator of cartilage breakdown (56, 57).

Comparison of the H-TS and the P-TS classes revealed an elevated level of lactate in pathological tendon sheaths; this trend could be explained by the conversion of pyruvate to lactate to maintain pentose-shunt activity during the inflammatory process (45). However, how this metabolite may play a role as a metabolic discriminator of pathology should be clarified with additional research. Concerning acetate, the same upward trend of pathological joints was also found for pathological tendon sheaths. The role of the other metabolites that we found in this study (acetylcholine and creatine) as indicators of pathological processes requires further investigation.

In addition, particular mention is necessary for two SF synovitis-affected samples from the P-TS group. Completely unexpectedly, these two samples show a similar metabolic profile to the OA/OCD-affected samples; however, in our opinion, it would be necessary to analyze more SF samples to confidently assume that synovitis exhibits the same metabolic pattern as OA and OCD.

In the present study, metabolomic analysis performed by ^1^H-NMR was used to identify and quantify a variety of biomarkers in SF. Based on both quantitative and qualitative information on the composition of SF from the anatomical sites considered, it was possible to distinguish healthy from pathological synovial fluids. Furthermore, the detection of these specific biomarkers allows the metabolic fingerprint of healthy and OA/OCD-affected joints and healthy and tenosynovitis-affected tendon sheaths to be established, fulfilling the aim of this study. Therefore, our hypothesis that the ^1^H-NMR-based metabolomic analysis is a valuable tool for analyzing the metabolite profile of normal and pathological joints and normal and pathological tendon sheaths of horses has been successfully satisfied.

However, a potential limitation of this study is the small sample size considered, especially for tendon sheaths; in our opinion, further SF samples of healthy and pathological joints and tendon sheaths would be needed to implement metabolomics data.

In our investigation, the analysis focused on distinguishing healthy from pathological samples, without differentiating between individual joint locations. This represents a limitation, as potential site-specific metabolic variability could influence the observed clustering patterns and metabolite concentrations. The metabolomic profile of joint synovial fluid may indeed vary according to the anatomical site from which it is collected, reflecting differences in joint biomechanics, cartilage composition, and local metabolic activity (58–60). Although anatomical heterogeneity of the sampling sites could still affect metabolomic patterns, the consistent application of this criterion across all groups and the clear clustering observed in multivariate analyses suggest that the differences identified primarily reflect pathological status rather than anatomical location. Further investigations involving larger cohorts and site-specific metabolomic characterization of both healthy and pathological joints are warranted to determine whether distinct metabolic fingerprints exist for each anatomical region.

To our knowledge, this is the first study that investigates the metabolic panel of healthy and pathological tendon sheaths using ^1^H-NMR. Thus, the results of our research support the results of previous studies in the field of metabolomics with ^1^H-NMR of healthy and OA-affected joints (35, 36) and provide new insights into the metabolomic analysis of healthy and tenosynovitis-affected tendon sheaths. These studies can provide an important contribution to clinical research as they provide a method to identify OA early, to investigate the progression of the disease, and to establish effective therapeutic treatment, reducing costs due to premature retirement of athletic subjects from competition. However, further studies are needed to implement the application of metabolomic analysis of tendon sheaths in the clinical field.

Furthermore, our study offers a valuable contribution to research on animals as a translational model for human osteoarthritis. The advantages of equine models for the translational study of human OA are the availability of abundant tissue to be collected, the possibility of arthroscopic investigations, and the ability to perform controlled training; whereas, the reported disadvantages are the personnel needed for safety and the need for special facilities (61). Therefore, in our opinion, our results could support research in the field of investigating the processes that characterize the synovial fluid metabolome and testing the effectiveness of diagnostic and therapeutic technologies in human medicine.

## 5 Conclusion

In this study, synovial fluid from joints of both healthy horses and OA- and OCD-affected horses, as well as from healthy and pathological tendon sheaths, was investigated.

Metabolomic analysis of the synovial fluid samples indicated that healthy joints and tendon sheaths share similar metabolic profiles, dominated by aerobic glycolysis, in which catabolic and anabolic pathways are balanced. In contrast, the metabolite profiles of pathological joints differed from those of healthy joints, and similarly, the metabolites of pathological tendon sheaths differed from those of healthy ones. These differences likely reflect homeostasis equilibrium loss caused by the increased catabolic processes that occur during inflammation.

To our knowledge, no studies have reported the metabolomic analysis of tendon sheath metabolites using ^1^H-NMR. Therefore, the present study contributes to expanding current knowledge on joint biomarkers in horses with osteoarthritis and provides a starting point for investigating biomarkers in tenosynovitis through a metabolomic approach. Applications of this research in the clinical field could facilitate an early diagnosis and enable objective evaluation of the effectiveness of treatments for joint and tendon sheath diseases. Furthermore, studying spontaneous models of joint and tendon injuries is particularly relevant, as it offers a realistic translational model of disease.

## Data Availability

The raw data supporting the conclusions of this article will be made available by the authors, without undue reservation.
