# Supplementary material for: Metabolomic analysis of synovial fluid from healthy and pathological equine joints and tendon sheaths using high-resolution 1H Nuclear Magnetic Resonance
Source: Front Vet Sci. 2025 Dec 16;12:1671176. doi: 10.3389/fvets.2025.1671176 (PMC12747927; doi:10.3389/fvets.2025.1671176)
Supplement: Supplementary file 1 [file Table_1.docx]

**LIST OF LEGENDS FOR SUPPLEMENTARY ITEMS**

| **Metabolite** | **Binned NMR signal**  **(ppm)** | **t.stat** | ***p*‐value** | **FDR (False Discovery Rate)** |
| --- | --- | --- | --- | --- |
| Acetate | 1.90 | -5.6965 | 3.6735E-6 | 1.3298E-4 |
| Glutamine | 2.46 | -8.9509 | 7.6626E-10 | 1.3869E-7 |
| Methionine | 2.12 | -6.8502 | 1.5934E-7 | 7.2101E-6 |
| Lactate | 1.34 | 3.6247 | 0.0010966 | 0.011648 |
| Pyruvate | 2.36 | 5.164 | 1.6077E-5 | 4.85E-4 |

**Table S1**. Univariate analysis (two samples t test) on the mean values +/- standard deviation of selected bucket reduced unbiased NMR signals after spectra normalization for the normal (H-J) vs the pathological (P-J) joint class samples.

| **Metabolite** | **Binned NMR signal**  **(ppm)** | **t.stat** | ***p*−value** | **FDR (False discovery rate)** |
| --- | --- | --- | --- | --- |
| Acetate | 1.90 | -4.3264 | 0.04712 | 0.04712 |
| Lactate | 1.34 | -2.0977 | 7.6626E-10 | 1.3869E-7 |
| α-Glucose | 5.22 | 3.1012 | 0.0050336 | 0.0075504 |
| β-Glucose | 4.62 | 4.0423 | 5.0645E-4 | 0.0010281 |

**Table S2.** Univariate analysis (two samples t test) on the mean values +/- standard deviation of selected bucket reduced unbiased NMR signals after spectra normalization for the normal (H-TS) vs the pathological (P-TS) tendon sheath class samples.
